# 5-Chloro-2-hy­droxy­benzaldehyde 4-ethyl­thio­semicarbazone

**DOI:** 10.1107/S160053681101796X

**Published:** 2011-05-20

**Authors:** Kong Mun Lo, Seik Weng Ng

**Affiliations:** a Department of Chemistry, University of Malaya, 50603 Kuala Lumpur, Malaysia

## Abstract

In the title compound, C_10_H_12_ClN_3_OS, the –C=N–N–C– chain bridging the ethyl­imino group and the benzene ring adopts an extended conformation with a C—N—N—C torsion angle of −171.98 (11)°. The imino H atom of the chain is a hydrogen-bond donor to the S atom of an inversion-related mol­ecule, forming a supra­molecular dimer. The hy­droxy H atom is intra­molecularly hydrogen bonded to the azomethine N atom.

## Related literature

For the salicyl­aldehyde 4-methyl­thio­semicarbazone homolog, see: Vrdoljak *et al.* (2005 ▶).
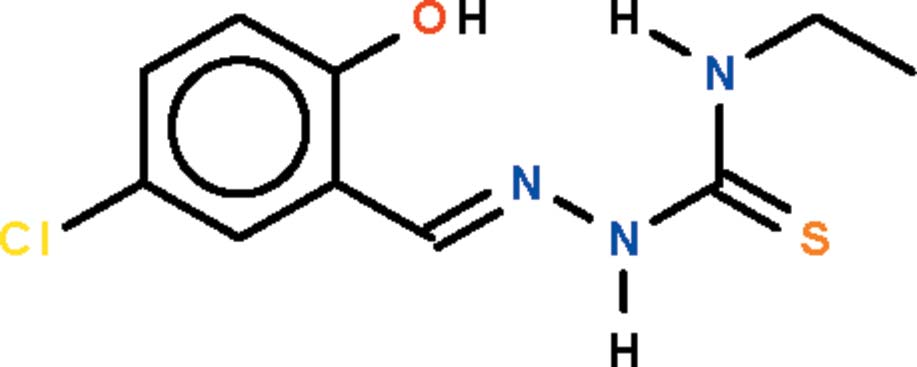


## Experimental

### 

#### Crystal data

C_10_H_12_ClN_3_OS
*M* *_r_* = 257.74
Monoclinic, 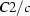

*a* = 21.7956 (3) Å
*b* = 11.8536 (2) Å
*c* = 9.4155 (1) Å
β = 101.6870 (9)°
*V* = 2382.12 (6) Å^3^
*Z* = 8
Mo *K*α radiation
μ = 0.48 mm^−1^
*T* = 100 K
0.40 × 0.40 × 0.40 mm

#### Data collection

Bruker SMART APEX diffractometer
Absorption correction: multi-scan (*SADABS*; Sheldrick, 1996 ▶) *T*_min_ = 0.832, *T*_max_ = 0.832
11204 measured reflections
2985 independent reflections
2582 reflections with *I* > 2σ(*I*)
*R*_int_ = 0.022

#### Refinement

*R*[*F* ^2^ > 2σ(*F* ^2^)] = 0.029
*wR*(*F* ^2^) = 0.085
*S* = 1.02
2985 reflections
157 parameters
3 restraints
H atoms treated by a mixture of independent and constrained refinement
Δρ_max_ = 0.33 e Å^−3^
Δρ_min_ = −0.28 e Å^−3^

### 

Data collection: *APEX2* (Bruker, 2009 ▶); cell refinement: *SAINT* (Bruker, 2009 ▶); data reduction: *SAINT*; program(s) used to solve structure: *SHELXS97* (Sheldrick, 2008 ▶); program(s) used to refine structure: *SHELXL97* (Sheldrick, 2008 ▶); molecular graphics: *X-SEED* (Barbour, 2001 ▶); software used to prepare material for publication: *publCIF* (Westrip, 2010 ▶).

## Supplementary Material

Crystal structure: contains datablocks global, I. DOI: 10.1107/S160053681101796X/xu5210sup1.cif

Structure factors: contains datablocks I. DOI: 10.1107/S160053681101796X/xu5210Isup2.hkl

Supplementary material file. DOI: 10.1107/S160053681101796X/xu5210Isup3.cml

Additional supplementary materials:  crystallographic information; 3D view; checkCIF report

## Figures and Tables

**Table 1:** Hydrogen-bond geometry (Å, °)

*D*—H⋯*A*	*D*—H	H⋯*A*	*D*⋯*A*	*D*—H⋯*A*
O1—H1o⋯N1	0.84 (1)	1.92 (1)	2.670 (2)	149 (2)
N2—H2n⋯S1^i^	0.87 (1)	2.48 (1)	3.308 (1)	159 (1)

Symmetry code: (i) 
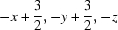
.

**Fig. 1.**
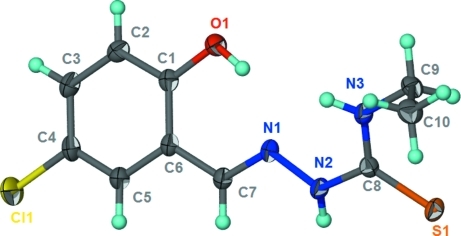
Thermal ellipsoid plot (Barbour, 2001) of C10H12ClN3OS at the 70% probability level; hydrogen atoms are drawn as spheres of arbitrary radius.

## supplementary crystallographic information

### Comment

Salicylaldehyde condenses with a large number of 4-alkyl/aryl-3-thiosemicarbazide to yield the corresponding thiosemicarbazone Schiff-bases. These compounds are used as chelating ligands to a range of metal ions. The semicarbazones, as exemplified by the salicylaldehyde 4-methyl-3-thiosemicarbazone homolog (Vrdoljak *et al.*, 2005), feature an N–H···S hydrogen bond that connects two molecules into a hydrogen-bonded dimer. In C_10_H_12_ClN_3_OS, the –C═N–N–C– chain separating the double-bond S atom and the benzene ring adopts an extended zigzag conformation (Fig.1). The amino H atom of the chain is hydrogen-bond donor to the S atom of an inversion-related molecule to form a dimer. The H atom of the hydroxy unit is hydrogen bond donor to the azomethine N atom. The other amino H atom is only weakly involved in hydrogen bonding (Table 1).

### Experimental

5-Chloro-2-hydroxybenzaldehyde (3.1 g, 20 mol) and of 4-ethyl-3-thiosemicarbazide (2.4 g, 20 mmol) were heated in ethanol (100 ml) for an hour. The solution was filtered and colorless crystals were obtained upon slow evaporation of the solvent.

### Refinement

Carbon-bound H-atoms were placed in calculated positions (C—H 0.95 to 0.99 Å) and were included in the refinement in the riding model approximation, with U_iso_(H) =1.5U_eq_(C) for methyl H atoms and 1.2U_eq_(C) for the others. The hydroxy and amino H atoms were located in a difference Fourier map, and were refined with distance restraints of O—H 0.84±0.01 and N—H 0.88±0.01 Å; their temperature factors were freely refined.

### Crystal data

**Table:**

C_10_H_12_ClN_3_OS	*F*(000) = 1072
*M**_r_* = 257.74	*D*_x_ = 1.437 Mg m^−^^3^
Monoclinic, *C*2/*c*	Mo *K*α radiation, λ = 0.71073 Å
Hall symbol: -C 2yc	Cell parameters from 6011 reflections
*a* = 21.7956 (3) Å	θ = 2.8–28.3°
*b* = 11.8536 (2) Å	µ = 0.48 mm^−^^1^
*c* = 9.4155 (1) Å	*T* = 100 K
β = 101.6870 (9)°	Block, colorless
*V* = 2382.12 (6) Å^3^	0.40 × 0.40 × 0.40 mm
*Z* = 8	

### Data collection

**Table:**

Bruker SMART APEX diffractometer	2985 independent reflections
Radiation source: fine-focus sealed tube	2582 reflections with *I* > 2σ(*I*)
graphite	*R*_int_ = 0.022
ω scans	θ_max_ = 28.4°, θ_min_ = 1.9°
Absorption correction: multi-scan (*SADABS*; Sheldrick, 1996)	*h* = −29→29
*T*_min_ = 0.832, *T*_max_ = 0.832	*k* = −15→15
11204 measured reflections	*l* = −12→12

### Refinement

**Table:**

Refinement on *F*^2^	Primary atom site location: structure-invariant direct methods
Least-squares matrix: full	Secondary atom site location: difference Fourier map
*R*[*F*^2^ > 2σ(*F*^2^)] = 0.029	Hydrogen site location: inferred from neighbouring sites
*wR*(*F*^2^) = 0.085	H atoms treated by a mixture of independent and constrained refinement
*S* = 1.02	*w* = 1/[σ^2^(*F*_o_^2^) + (0.0483*P*)^2^ + 1.7745*P*] where *P* = (*F*_o_^2^ + 2*F*_c_^2^)/3
2985 reflections	(Δ/σ)_max_ = 0.001
157 parameters	Δρ_max_ = 0.33 e Å^−^^3^
3 restraints	Δρ_min_ = −0.28 e Å^−^^3^

### Fractional atomic coordinates and isotropic or equivalent isotropic displacement parameters (Å^2^)

**Table:**

	*x*	*y*	*z*	*U*_iso_*/*U*_eq_	
Cl1	0.485785 (16)	0.65980 (4)	0.51634 (4)	0.03990 (13)	
S1	0.835591 (14)	0.65767 (3)	−0.01381 (3)	0.01821 (10)	
O1	0.75861 (4)	0.62629 (8)	0.57947 (10)	0.01950 (19)	
N1	0.73919 (5)	0.61554 (8)	0.29057 (11)	0.0159 (2)	
N2	0.75536 (5)	0.63100 (9)	0.15711 (11)	0.0167 (2)	
N3	0.85219 (5)	0.55435 (9)	0.24435 (11)	0.0169 (2)	
C1	0.69537 (6)	0.62896 (10)	0.56025 (13)	0.0160 (2)	
C2	0.66935 (6)	0.63906 (10)	0.68348 (14)	0.0189 (2)	
H2	0.6959	0.6406	0.7769	0.023*	
C3	0.60511 (6)	0.64685 (11)	0.67054 (14)	0.0215 (3)	
H3	0.5875	0.6549	0.7544	0.026*	
C4	0.56661 (6)	0.64280 (12)	0.53368 (15)	0.0230 (3)	
C5	0.59099 (6)	0.63056 (11)	0.41045 (14)	0.0203 (3)	
H5	0.5638	0.6262	0.3180	0.024*	
C6	0.65605 (5)	0.62453 (10)	0.42189 (13)	0.0160 (2)	
C7	0.68017 (5)	0.62429 (10)	0.28864 (13)	0.0166 (2)	
H7	0.6517	0.6307	0.1982	0.020*	
C8	0.81461 (5)	0.61070 (10)	0.14031 (12)	0.0152 (2)	
C9	0.91574 (5)	0.51980 (11)	0.23606 (13)	0.0199 (2)	
H9A	0.9414	0.5132	0.3353	0.024*	
H9B	0.9350	0.5783	0.1840	0.024*	
C10	0.91595 (6)	0.40780 (12)	0.15821 (14)	0.0235 (3)	
H10A	0.9591	0.3872	0.1542	0.035*	
H10B	0.8911	0.4144	0.0594	0.035*	
H10C	0.8977	0.3494	0.2107	0.035*	
H1O	0.7673 (10)	0.6201 (18)	0.4975 (14)	0.057 (6)*	
H2N	0.7319 (6)	0.6770 (11)	0.0975 (14)	0.017 (4)*	
H3N	0.8351 (7)	0.5207 (13)	0.3075 (15)	0.026 (4)*	

### Atomic displacement parameters (Å^2^)

**Table:**

	*U*^11^	*U*^22^	*U*^33^	*U*^12^	*U*^13^	*U*^23^
Cl1	0.01591 (17)	0.0783 (3)	0.0285 (2)	0.00085 (16)	0.01169 (14)	0.00069 (17)
S1	0.01839 (16)	0.02150 (16)	0.01680 (15)	0.00297 (10)	0.00847 (12)	0.00232 (11)
O1	0.0152 (4)	0.0239 (5)	0.0195 (4)	0.0018 (3)	0.0039 (3)	0.0007 (4)
N1	0.0165 (5)	0.0162 (5)	0.0165 (5)	0.0003 (4)	0.0072 (4)	0.0002 (4)
N2	0.0151 (5)	0.0210 (5)	0.0152 (5)	0.0030 (4)	0.0061 (4)	0.0030 (4)
N3	0.0144 (4)	0.0207 (5)	0.0166 (5)	0.0012 (4)	0.0055 (4)	0.0015 (4)
C1	0.0163 (5)	0.0135 (5)	0.0189 (5)	0.0006 (4)	0.0054 (4)	0.0010 (4)
C2	0.0218 (6)	0.0191 (6)	0.0163 (5)	−0.0002 (4)	0.0047 (5)	−0.0004 (4)
C3	0.0241 (6)	0.0238 (6)	0.0193 (6)	−0.0001 (5)	0.0106 (5)	0.0001 (5)
C4	0.0152 (6)	0.0318 (7)	0.0241 (6)	−0.0007 (5)	0.0092 (5)	0.0011 (5)
C5	0.0172 (6)	0.0263 (6)	0.0183 (6)	−0.0019 (5)	0.0053 (5)	0.0013 (5)
C6	0.0163 (5)	0.0162 (5)	0.0170 (5)	−0.0007 (4)	0.0070 (4)	0.0003 (4)
C7	0.0164 (5)	0.0175 (5)	0.0167 (5)	−0.0009 (4)	0.0053 (4)	0.0006 (4)
C8	0.0148 (5)	0.0157 (5)	0.0159 (5)	−0.0010 (4)	0.0047 (4)	−0.0031 (4)
C9	0.0127 (5)	0.0251 (6)	0.0217 (6)	0.0022 (4)	0.0031 (4)	0.0000 (5)
C10	0.0185 (6)	0.0273 (7)	0.0248 (6)	0.0043 (5)	0.0046 (5)	−0.0015 (5)

### Geometric parameters (Å, °)

**Table:**

Cl1—C4	1.7476 (13)	C2—H2	0.9500
S1—C8	1.7011 (12)	C3—C4	1.3889 (19)
O1—C1	1.3539 (14)	C3—H3	0.9500
O1—H1O	0.835 (9)	C4—C5	1.3782 (18)
N1—C7	1.2870 (15)	C5—C6	1.4020 (16)
N1—N2	1.3842 (13)	C5—H5	0.9500
N2—C8	1.3541 (14)	C6—C7	1.4552 (16)
N2—H2N	0.870 (9)	C7—H7	0.9500
N3—C8	1.3234 (15)	C9—C10	1.5169 (18)
N3—C9	1.4615 (14)	C9—H9A	0.9900
N3—H3N	0.860 (9)	C9—H9B	0.9900
C1—C2	1.3959 (17)	C10—H10A	0.9800
C1—C6	1.4078 (17)	C10—H10B	0.9800
C2—C3	1.3837 (17)	C10—H10C	0.9800
			
C1—O1—H1O	107.2 (15)	C6—C5—H5	120.1
C7—N1—N2	114.46 (10)	C5—C6—C1	119.07 (11)
C8—N2—N1	120.45 (10)	C5—C6—C7	118.05 (11)
C8—N2—H2N	119.1 (10)	C1—C6—C7	122.65 (11)
N1—N2—H2N	116.3 (10)	N1—C7—C6	121.50 (11)
C8—N3—C9	123.58 (10)	N1—C7—H7	119.2
C8—N3—H3N	117.1 (11)	C6—C7—H7	119.2
C9—N3—H3N	117.0 (11)	N3—C8—N2	117.72 (10)
O1—C1—C2	117.70 (11)	N3—C8—S1	124.30 (9)
O1—C1—C6	122.36 (11)	N2—C8—S1	117.97 (9)
C2—C1—C6	119.92 (11)	N3—C9—C10	111.51 (10)
C3—C2—C1	120.44 (12)	N3—C9—H9A	109.3
C3—C2—H2	119.8	C10—C9—H9A	109.3
C1—C2—H2	119.8	N3—C9—H9B	109.3
C2—C3—C4	119.33 (12)	C10—C9—H9B	109.3
C2—C3—H3	120.3	H9A—C9—H9B	108.0
C4—C3—H3	120.3	C9—C10—H10A	109.5
C5—C4—C3	121.41 (12)	C9—C10—H10B	109.5
C5—C4—Cl1	119.13 (10)	H10A—C10—H10B	109.5
C3—C4—Cl1	119.40 (10)	C9—C10—H10C	109.5
C4—C5—C6	119.81 (12)	H10A—C10—H10C	109.5
C4—C5—H5	120.1	H10B—C10—H10C	109.5
			
C7—N1—N2—C8	−171.98 (11)	C2—C1—C6—C5	0.09 (17)
O1—C1—C2—C3	177.38 (11)	O1—C1—C6—C7	−4.18 (17)
C6—C1—C2—C3	−1.19 (18)	C2—C1—C6—C7	174.32 (12)
C1—C2—C3—C4	0.97 (19)	N2—N1—C7—C6	−172.18 (10)
C2—C3—C4—C5	0.4 (2)	C5—C6—C7—N1	−177.90 (11)
C2—C3—C4—Cl1	−176.77 (10)	C1—C6—C7—N1	7.82 (18)
C3—C4—C5—C6	−1.5 (2)	C9—N3—C8—N2	175.63 (11)
Cl1—C4—C5—C6	175.68 (10)	C9—N3—C8—S1	−3.54 (17)
C4—C5—C6—C1	1.21 (18)	N1—N2—C8—N3	13.77 (17)
C4—C5—C6—C7	−173.28 (12)	N1—N2—C8—S1	−167.01 (8)
O1—C1—C6—C5	−178.40 (11)	C8—N3—C9—C10	−85.80 (14)

### Hydrogen-bond geometry (Å, °)

**Table:**

*D*—H···*A*	*D*—H	H···*A*	*D*···*A*	*D*—H···*A*
O1—H1o···N1	0.84 (1)	1.92 (1)	2.670 (2)	149 (2)
N2—H2n···S1^i^	0.87 (1)	2.48 (1)	3.308 (1)	159 (1)

Symmetry codes: (i) −*x*+3/2, −*y*+3/2, −*z*.
